# Climate‐driven mitochondrial selection in lacertid lizards

**DOI:** 10.1002/ece3.11176

**Published:** 2024-03-24

**Authors:** Xiang Zhang, Jian Chen, Hong‐Yu Luo, Xin Chen, Jun Zhong, Xiang Ji

**Affiliations:** ^1^ Zhejiang Provincial Key Laboratory for Water Environment and Marine Biological Resources Protection, College of Life and Environmental Sciences Wenzhou University Wenzhou China

**Keywords:** climatic variables, gene arrangement, Lacertidae, mitochondrial genomes, phylogeny, positive selection gene

## Abstract

The mitochondrion, which is an intracellular organelle responsible for most of the energy‐producing pathways, can have its genome targeted for climate‐driven selection. However, climate‐driven mitochondrial selection remains a sparsely studied area in reptiles. Here, we reported the complete mitochondrial genome sequence of a lacertid lizard (*Takydromus intermedius*) and used mitogenomes from 54 species of lacertid lizards to study their phylogenetic relationships and to identify the mitochondrial genes under positive selection by climate. The length of the complete mitochondrial genome sequence of *T. intermedius* was 17,713 bp, which was within the range of lengths (17,224–18,943) ever reported for *Takydromus* species. The arrangement of mitochondrial genes in *T. intermedius* was the same as in other congeneric species. The 54 lacertid species could be divided into three geographically and climatically different clades. We identified three mitochondrial genes (*ATP*6, *ATP8*, and *ND3*) under positive selection by climate, and found that isothermality, temperature seasonality, precipitation of wettest month, and precipitation seasonality were the most important climatic variables contributing to the gene selection.

## Graphical abstract

We investigated the mitogenoms of *Takydromus intermedius*. We found three genes (*ATP6*, *ATP8*, and *ND3*) positively selected by climate inlizards.

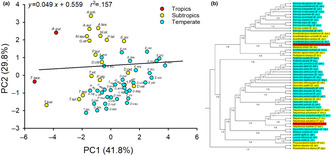

## 1 INTRODUCTION

Long‐term natural selection leads to genetic evolution, which in turn promotes divergence between populations and ultimately drives speciation (Lamb et al., 2018; Volis et al., 2019). Climate change is one of main pressures for organisms, shaping behavioral, and physiological mechanisms involved in animal adaptation to environmental challenges (Dupoue et al., 2017; Franks & Hoffmann, 2012; Kovac et al., 2020). Climate‐driven selection on genes and phenotypic traits contributes greatly to local adaptation and intraspecific differentiation in animals (Guo, Zhong, Xie, et al., 2021; Guo, Zhong, Zhu, et al., 2021; Kovac et al., 2020; Olson et al., 2013; Sikdar, 2023). The mitochondrion, which is an intracellular organelle in eukaryotic cells responsible for most of the energy‐producing pathways (Chong & Mueller, 2013), can have its genome targeted for climate‐driven selection (Lamb et al., 2018; Scott et al., 2011; Sun et al., 2018). The mitochondrial density and respiration rate often vary among populations or species from different climates (Pichaud et al., 2020; White et al., 2012).

Varying among species in size and gene arrangement (Lamb et al., 2018; Scott et al., 2011), mitochondrial genomes (mitogenomes) have been extensively used to infer phylogenetic relationships and evolutionary processes. The mitogenome is typically a closed circular double‐stranded DNA molecule, often 15–20 kb in length and coding 37 genes, including 22 transfer RNA (tRNA) genes, 13 protein‐coding genes (PCGs), and two ribosomal RNA (rRNA) genes; it also includes a noncoding (control) region of variable length that plays a regulatory role in transcription and replication (Boore, 1999). The 13 proteins encoded by mitochondrial PCGs are essential components of the enzymes involved in the citric acid cycle and oxidative phosphorylation (OXPHOS) pathway (Ballard & Pichaud, 2014; Chong & Mueller, 2013; Zhang et al., 2021). Mitochondrial genes may experience different selection pressures among populations or species that differ in distribution or use different habitats. For instance, variation in amino acids at certain specific sites of cytochrome c oxidase (COX) proteins encoded by mitochondrial genes may enhance mitochondrial respiration during hypoxia, and is therefore considered to be related to high‐altitude adaptation in animals (Luo et al., 2008; Xu et al., 2005). In humans, *ATP6*, *Cytb*, *COX1*, and *ND4* have experienced natural selection by climate (Balloux et al., 2009; Mishmar et al., 2003; Zhang et al., 2013). Climate induces the diversifying selection on *ND4* and *ATP6* in two *Tetranychus* species of spider mites (Sun et al., 2018). Climate drives the gene selection of *ND5*, *ND6*, and *COX1* in Australian songbirds (Lamb et al., 2018). Earlier studies consistently suggest that mitochondrial genes are susceptible to climate change.

Lacertidae is not a very species‐rich family, currently including 44 genera and some 370 species (http://reptile‐database.reptarium.cz/; Arnold et al., 2007). The family has been divided into two subfamilies, Gallotiinar and Lacertinae, with the latter subfamily further divided into two clades (Arnold et al., 2007; Pyron et al., 2013). Phylogenetic relationships within the family Lacertidae were once unclear because of the indistinguishable morphological characteristics such as the relationship between the genera *Takydromus* and *Platyplacopus* (Arnold, 1997). The phylogenetic analyses of the mitochondrial DNA (mtDNA) sequences have provided new insight into the evolutionary relationships within Lacertidae (Arnold et al., 2007; Chen et al., 2009; Guo et al., 2011). For example, the phylogeny inferred from cyt *b* gene supports the idea that the genus *Platyplacopus* should be merged into the genus *Takydromus* (Chen et al., 2009).

Lacertid lizards are widely distributed in Eurasia and Africa (Arnold et al., 2007), covering a range from temperate to tropical areas with a wide variety of climatic conditions that may induce geographic variation in metabolic rates and thermoregulation ability (Guo, Zhong, Xie, et al., 2021; Guo, Zhong, Zhu, et al., 2021; He et al., 2013). However, whether climatic differences may lead to differential selection pressures on the mtDNA in the family Lacertidae remains unknown. Here, we reported the complete mitochondrial genome sequence of a lacertid lizard (*Takydromus intermedius*) and used mitogenoms from 54 lacertid species to study their phylogenetic relationships and to identify the mitochondrial genes under positive selection by climate, aiming to investigate climate‐driven mitochondrial selection in lizards.

## 2 MATERIALS AND METHODS

### 2.1 Sample collection and DNA extraction

We collected an adult female of *T. intermedius* from Bashu (106°55′ N, 29°10′ E), Chongqing in July 2021. The lizard was brought to our laboratory at Wenzhou University, where the most distant 15 mm of its tail tip was excised using a sterilized scalpel. The lizard was allowed to heal the wound for 3 days after the tail‐excising event, and was thereafter kept under the laboratory conditions designed specifically for *Takydromus* lizards (Guo, Zhong, Xie, et al., 2021; Guo, Zhong, Zhu, et al., 2021; Ji et al., 2007; Lin & Ji, 2005; Luo et al., 2010; Ma et al., 2019). The lizard died 8 months after it was collected. We preserved its body in 95% ethanol and stored it in our laboratory for possible use later. We used the DNeasy Tissue Kit (Qiagen, Germany) to extract total genomic DNA from muscle tissue of the tail sample, following the manufacturer's instructions.

### 2.2 Mitochondrial DNA amplification and sequence analysis

We designed 15 pairs of primers (Table S1) to amplify contiguous and overlapping fragments of the complete mitochondrial genome, following the procedures described in earlier studies of other *Takydromus* species. The amplifications were performed with Taq DNA polymerase (TaKaRa, Dalian, China) in a PCR Instrument (Biometra Tone 96, Jena, Germany). The products were sequenced by Sangon Biotech (Shanghai) Co., Ltd. The PCR process and sequencing were repeated twice to ensure the accuracy of the results.

The mitogenome sequence was manually aligned and corrected using the Conting Express 9.1. We used the MITOS (http://mitos.bioinf.uni‐leipzig.de/index.py) online server (Kumar et al., 2016; Lamb et al., 2018) to locate the position of each of 37 genes (13 protein‐coding genes, two rRNA genes and 22 tRNA genes). The MITOS was also used to identify the tRNA secondary structures and positions according to that in other lizards. The start and end codons of all 13 PCGs were tested in Open Reading Frame Finder via NCBI (https://www.ncbi.nlm.nih.gov/orffinder/) and MITOS based on the annotated mitogenome of *T. amurensis*.

### 2.3 Phylogenetic analysis

We downloaded the mitogenome sequences of other 53 lacertid species from the NCBI database to analyze phylogenetic relationships within Lacertidae (Table S2). We used MrBayes 3.2.6 under GTR + I + G + F (50,000,000 generations) with the initial 25% burn‐in (Ronquist et al., 2012) to construct the Bayesian Inference (BI) tree for the 56 species, including 54 lacertid species and two gekkonid species of the genus *Gekko* (*G. subpalmatus* and *G. hokounesis*), of which the latter two were used as outgroups (Table S2). The nucleotide sequences of 13 PCGs and two rRNAs (12S rRNA and 16S rRNA) were used in phylogenetic analysis and aligned by MUSCLE codon alignment implemented in MEGA 7 (Kumar et al., 2016).

### 2.4 Climatic variables

We used the point sampling tool in DIVA GIS to extract values for each climatic variable of the distribution points from the 54 lacertid species with a spatial resolution of 30 arc seconds (~1 km) from raster layers in WorldClim 1.4. (www.worldclim.org/current). Nineteen climatic variables were used to represent the climatic drivers of selection (Guo, Zhong, Xie, et al., 2021; Guo, Zhong, Zhu, et al., 2021). According to the climatic variables >50% distribution areas, we assigned the habitats of the 54 species of lacertid lizards into one of three (tropic, subtropic, and temperate) groups. The 19 climatic variables were standardized within the range from −1 to 1 before they were used in a principal components analysis (PCA) for climatic classification of the habitats. We used R 3.6.1 to perform phylogenetic generalized least squares (PGLS) regressions in Caper 1.0.1 package (Orme et al., 2018) and thereby tested whether climatic classification of the habitats was consistent with phylogenetic relationships within Lacertidae.

### 2.5 Molecular evolution analyses

We used phylogenetic analysis by maximum likelihood in EasyCodeML to estimate the ratio of nonsynonymous to synonymous substitution ratios (*ω* = *d*N/*d*S) and to analyze the episodic gene selection on particular sites in mitogenomes in a climatic category (Gao et al., 2019; Weadick & Chang, 2012; Yang, 2007). The *ω* is a widely used as a measure of gene selection, with *ω* <1, =1, and >1, respectively, representing purifying selection, neutral selection, and positive selection (Kimura, 1979; Xia et al., 2019). The Clade model is usually used to accommodate site‐specific divergence in selective constraint for two or more clades in the tree (Forsberg & Christiansen, 2003). We chose the most commonly used Clade model C (CmC) to estimate separate *ω* ratios along particular branches (Weadick & Chang, 2012). Based on the topology analysis of the 54 lacertid lizards, we used Clade model to evaluate the evolutionary rate for each branch, assigning *T. intermedius* as foreground and the others as background branches. The commonly used paired models of CmC versus M2a‐rel (a model assuming under the same selection pressure) were used to test the significance of the selection in each climate extreme by likelihood ratio tests.

Using the R package gradientForest, we performed gradient forest analysis to detect the differences in positive selection effects of different climate factors on different genes (Ellis et al., 2012). To perform this analysis, we calculated genetic distances (p‐distances) of the positive selection genes and fitted them to 19 current climatic variables for the 54 lacertid species including *T. intermedius*. We then calculated Euclidian distances between the positive selection genes and the climatic variables. A greater Euclidian distance suggests a greater influence of a climatic variable on genetic distance.

## 3 RESULTS

The length of the complete mitochondrial genome sequence of *T. intermedius* was 17,713 bp (GenBank accession Number OQ632596), which was within the range of lengths (17,224–18,943) ever reported for *Takydromus* species (Table S3). The gene arrangement of 13 PCGs, 22 tRNAs, two rRNAs (12S rRNA and 16S rRNA), and one D‐loop region (2330 bp) in *T. intermedius* was the same as in other congeneric species. Nine genes, including *ND6* and eight tRNA genes (*tRNA‐Gln*, *tRNA‐Ala*, *tRNA‐Asn*, *tRNA‐Cys*, *tRNA‐Tyr*, *tRNA‐Ser*, *tRNA‐Glu*, and *tRNA‐Pro*), were on the light strand, and the others were on the heavy strand. The AT content (59.9%) of the mitogenome was smaller in *T. intermedius* than in other *Takydromus* species studied thus far (Table S3). The AT skew was 0.04 in *T. intermedius*, which was the same as in *T. amurensis* and higher than the values reported for other *Takydromus* species; the GC skew was −0.32 in *T. intermedius*, which was smaller than the values reported for other *Takydromus* species (Table S3). The 13 PCGs ranged in size from 162 bp (*Atp8*) to 1827 bp (*ND5*) and started with ATG (Table S4). The TAA was the most frequent stop codon, as revealed by the fact that, of the 13 PCGs, six (*ND1*, *ND3*, *ND4L*, *ND5*, *ATP6*, and *ATP8*), three (*COX2*, *COX3*, and *ND4*), two (*ND2* and *Cytb*), one (*COX1*), and one (*ND6*) ended with TAA, T, TAG, AGG, and AGA, respectively (Table S4).

**FIGURE 1:**
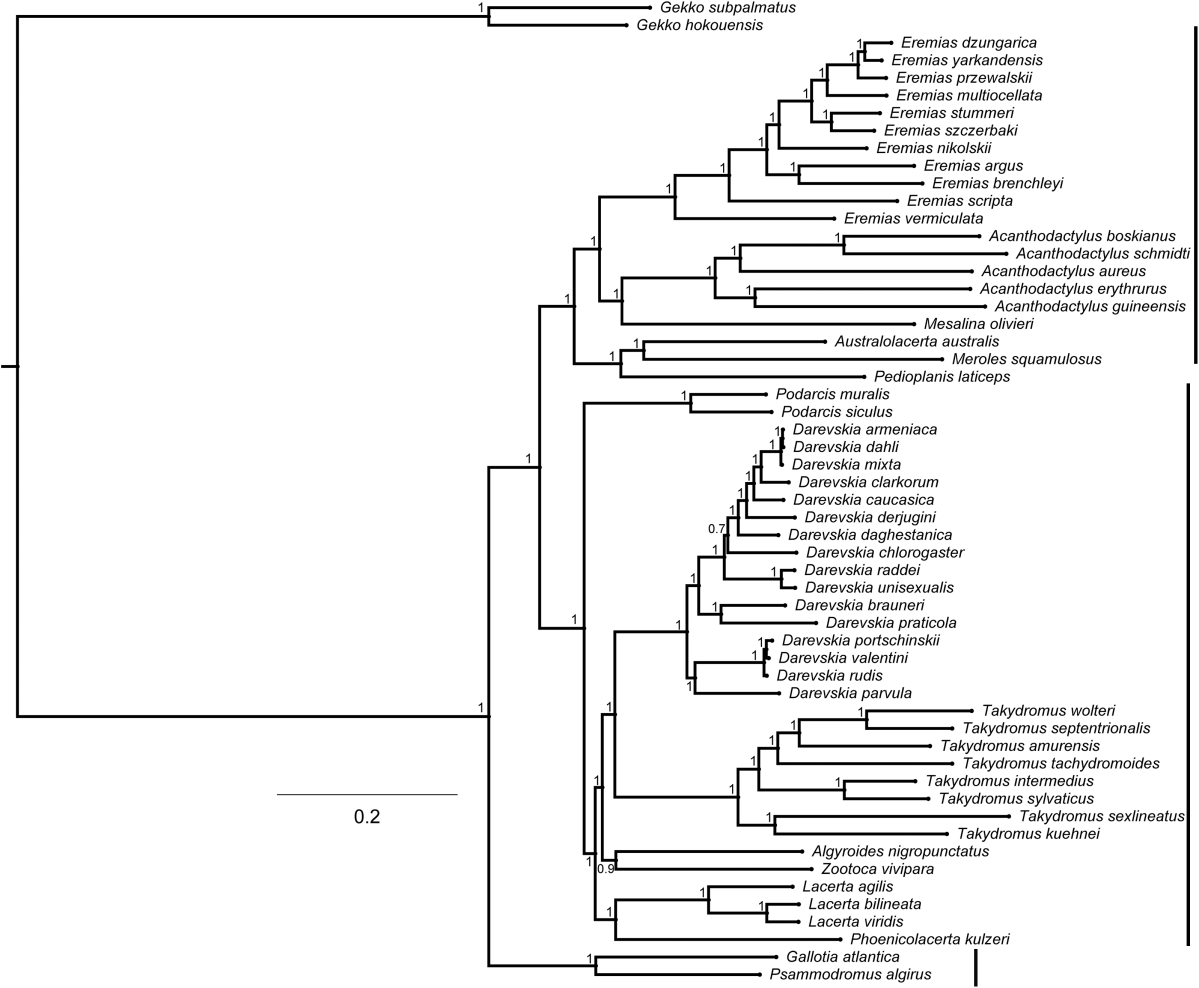
The Bayesian phylogenetic tree based on the mitochondrial genes (two rRNA genes and 13 PCGs) of 54 species of lacertid lizards. The model used is the MUSCLE codon. Value on each branch corresponds to the posterior probability obtained with the Bayesian inference analysis.

The 54 lacertid species could be divided into three main clades (Figure 1). More specifically, *Gallotia atlantica* and *Psammodromus algirus* formed a clade, *Algyroides nigropunctatus*, *Phoenicolacerta kulzeri*, *Zootoca vivipara*, and species of the genera *Darevskia*, *Lacerta*, *Podarcis*, and *Takydromus* formed a clade, and *Australolacerta australis*, *Mesalina olivieri*, *Meroles squamulosus*, and species of the genera *Acanthodactylus* and *Eremias* formed a clade (Figure 1). *Takydromus intermedius* was a sister taxon to *T. sylvaticus* (Figure 1).

**FIGURE 2:**
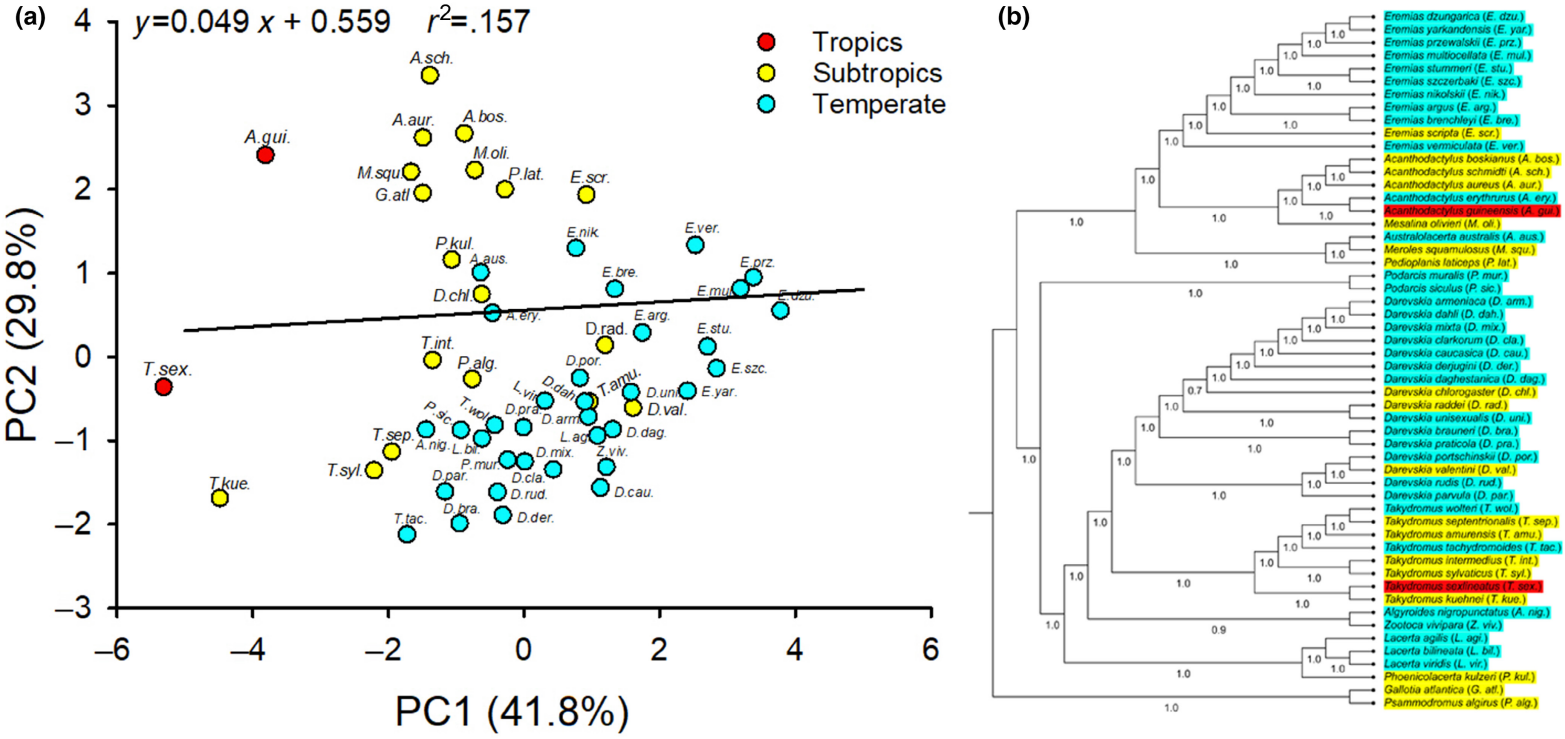
Positions of tropics, subtropics, and temperate species of lacertid lizards in the space defined by the first two axes of principal components analysis based on 19 climatic variables (a), and the phylogeny of the 54 species of lizards obtained from Bayesian inference analysis based on mitochondrial genes (b). Each color indicates a climate region.

PCA resolved two components (eigenvalues ≥1) from the 19 climatic variables for the 54 lacertid species, generally confirming the grouping of three climatic types (Figure 2a). PC1 and PC2 accounted for 41.8% and 29.8% of total variance in the original climatic data, respectively (Figure 2a). *Acanthodactylus guineensis* and *T. sexlineatus* could be assigned to the tropic group, 18 species including five *Takydromus* species could be assigned to the subtropic group, and the remaining 32 species mainly including *Eremias* and *Darevskia* species could be assigned to the temperate group (Figure 2a). The climatic factors of PC1 and PC2 exhibited strongest phylogenetic signal (*λ* = 0.869) (Figure 2b).

**FIGURE 3:**
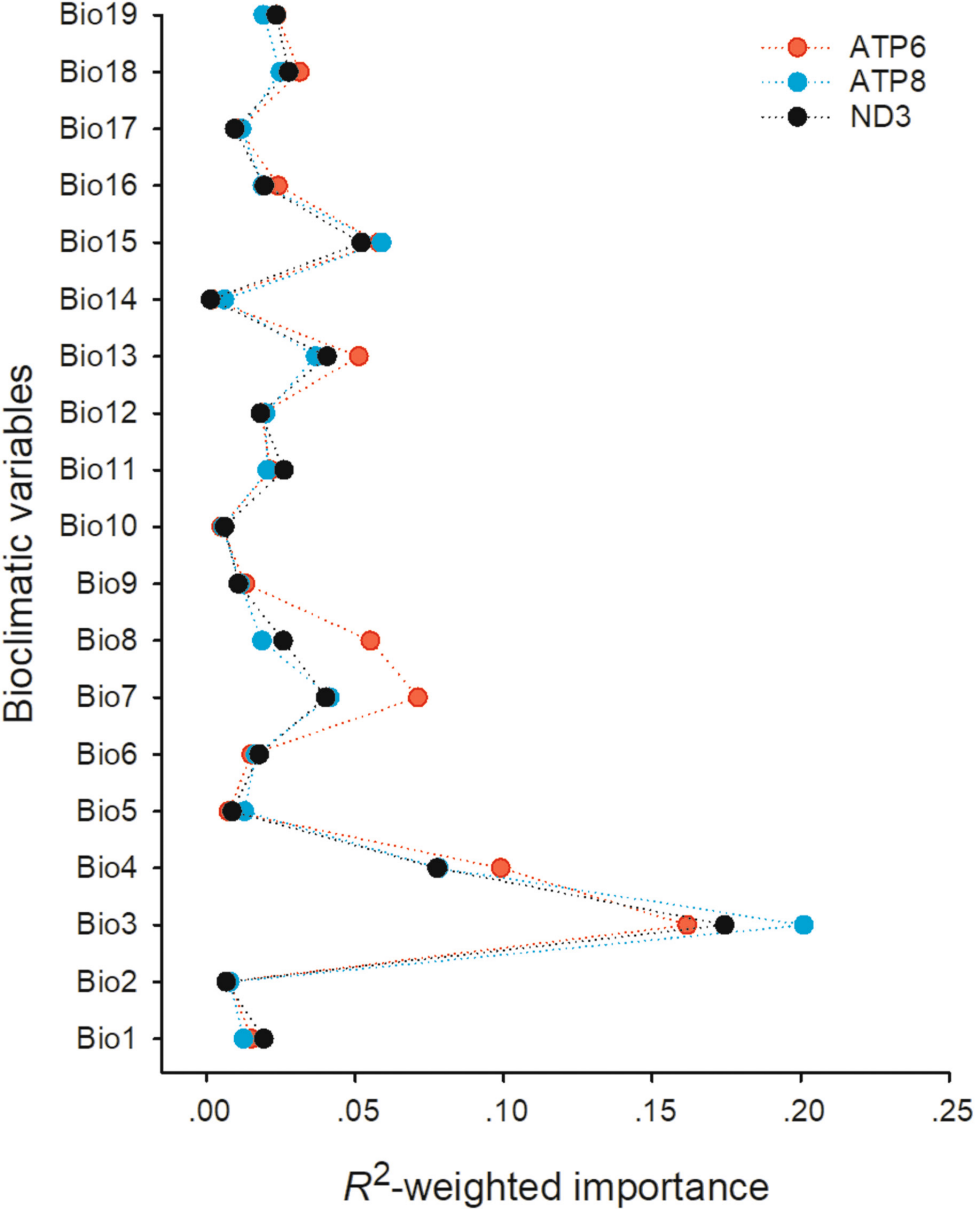
Results of the gradient forest analysis, showing the contribution of each climatic variable to the gene selection.

The CodeML CMC revealed five genes (*ND2*, *ATP*6, *ATP8*, *ND3*, and *ND4L*) as candidates for positive selection (*ω* > 1), with three (*ATP*6, *ATP8*, and *ND3*) confirmed by CodeML M2a‐rel (Likehood Ratio Test (LRT) *p* values <.01) (Table 1). Gradient forest analysis revealed that all three positive selection genes were affected by climatic variables, with *ATP6* being the most climatically sensitive gene (Figure 3). The top two explanatory climatic variables tested in gradient forest analysis were Bio3 (isothermality) and Bio4 (temperature seasonality). Two precipitation‐related variables, Bio13 (precipitation of wettest month) and Bio15 (precipitation seasonality), contributed significantly to variation in the three positively selected genes.

**TABLE 1:** Test for positive selection in divergent clades of 13 protein‐coding genes with clade model.

Genes	Models	Number of parameters	Log‐likelihhood values	Parameter estimates	LRT *p* values
*ND1*	CmC	114	−17,575.91	*ω*_0_ = 0.01, *p*_0_ = .69; *ω*_1_ = 1.00, *p*_1_ = .01; *ω*_2_ = 0.27, *p*_2_ = .30	.30
	M2a‐rel	111	−17,577.72	*ω*_0_ = 0.01, *p*_0_ = .68; *ω*_1_ = 1.00, *p*_1_ = .01; *ω*_2_ = 0.16, *p*_2_ = .30	
*ND2*	CmC	114	−14,547.02	*ω*_0_ = 0.04, *p*_0_ = .64; *ω*_1_ = 1.00, *p*_1_ = .02; *ω*_2_ = 1.70, *p*_2_ = .32	.17
	M2a‐rel	111	−14,549.54	*ω*_0_ = 0.04, *p*_0_ = .64; *ω*_1_ = 1.00, *p*_1_ = .026; *ω*_2_ = 0.30, *p*_2_ = .33	
*COX1*	CmC	114	−23,878.24	*ω*_0_ = 0.01, *p*_0_ = .78; *ω*_1_ = 1.00, *p*_1_ = .02; *ω*_2_ = 0.14, *p*_2_ = .21	.17
	M2a‐rel	111	−23,880.81	*ω*_0_ = 0.01, *p*_0_ = .77; *ω*_1_ = 1.00, *p*_1_ = .02; *ω*_2_ = 0.19, *p*_2_ = .22	
*COX2*	CmC	114	−10,951.66	*ω*_0_ = 0.01, *p*_0_ = .78; *ω*_1_ = 1.00, *p*_1_ = .02; *ω*_2_ = 0.99, *p*_2_ = .23	.97
	M2a‐rel	111	−10,952.16	*ω*_0_ = 0.01, *p*_0_ = .79; *ω*_1_ = 1.00, *p*_1_ = .03; *ω*_2_ = 0.13, *p*_2_ = .19	
ATP8*	CmC	114	−4027.80	*ω*_0_ = 0.01, *p*_0_ = .77; *ω*_1_ = 1.00, *p*_1_ = .00; *ω*_2_ = 2.10, *p*_2_ = .28	.00 **
	M2a‐rel	111	−3987.86	*ω*_0_ = 0.01, *p*_0_ = .71; *ω*_1_ = 1.00, *p*_1_ = .01; *ω*_2_ = 0.30, *p*_2_ = .28	
ATP6*	CmC	114	−15,706.23	*ω*_0_ = 0.05, *p*_0_ = .89; *ω*_1_ = 1.00, *p*_1_ = .11; *ω*_2_ = 1.35, *p*_2_ = .00	.00 **
	M2a‐rel	111	−15,346.91	*ω*_0_ = 0.01, *p*_0_ = .59; *ω*_1_ = 1.00, *p*_1_ = .07; *ω*_2_ = 0.35, *p*_2_ = .13	
*COX3*	CmC	114	−13,231.02	*ω*_0_ = 0.01, *p*_0_ = .87; *ω*_1_ = 1.00, *p*_1_ = .05; *ω*_2_ = 0.25, *p*_2_ = .12	.35
	M2a‐rel	111	−13,232.65	*ω*_0_ = 0.01, *p*_0_ = .87; *ω*_1_ = 1.00, *p*_1_ = .01; *ω*_2_ = 0.19, *p*_2_ = .12	
ND3*	CmC	114	−7091.81	*ω*_0_ = 0.01, *p*_0_ = .77; *ω*_1_ = 1.00, *p*_1_ = .00; *ω*_2_ = 2.10, *p*_2_ = .28	.00 **
	M2a‐rel	111	−7075.18	*ω*_0_ = 0.00, *p*_0_ = .56; *ω*_1_ = 1.00, *p*_1_ = .14; *ω*_2_ = 0.15, *p*_2_ = .29	
*ND4L*	CmC	114	−6561.82	*ω*_0_ = 0.03, *p*_0_ = .75; *ω*_1_ = 1.00, *p*_1_ = .00; *ω*_2_ = 1.42, *p*_2_ = .25	.16
	M2a‐rel	111	−6564.44	*ω*_0_ = 0.02, *p*_0_ = .40; *ω*_1_ = 1.00, *p*_1_ = .06; *ω*_2_ = 0.15, *p*_2_ = .55	
*ND4*	CmC	114	−29,617.84	*ω*_0_ = 0.02, *p*_0_ = .64; *ω*_1_ = 1.00, *p*_1_ = .03; *ω*_2_ = 0.39, *p*_2_ = .33	.14
	M2a‐rel	111	−29,620.54	*ω*_0_ = 0.02, *p*_0_ = .64; *ω*_1_ = 1.00, *p*_1_ = .03; *ω*_2_ = 0.21, *p*_2_ = .33	
*ND5*	CmC	114	−33,999.61	*ω*_0_ = 0.01, *p*_0_ = .61; *ω*_1_ = 1.00, *p*_1_ = .01; *ω*_2_ = 0.44, *p*_2_ = .38	.14
	M2a‐rel	111	−34,002.36	*ω*_0_ = 0.01, *p*_0_ = .61; *ω*_1_ = 1.00, *p*_1_ = .01; *ω*_2_ = 0.13, *p*_2_ = .38	
*ND6*	CmC	114	−9227.54	*ω*_0_ = 0.02, *p*_0_ = .62; *ω*_1_ = 1.00, *p*_1_ = .03; *ω*_2_ = 0.35, *p*_2_ = .36	.87
	M2a‐rel	111	−9229.57	*ω*_0_ = 0.02, *p*_0_ = .60; *ω*_1_ = 1.00, *p*_1_ = .03; *ω*_2_ = 0.24, *p*_2_ = .37	
*Cytb*	CmC	114	−19,637.30	*ω*_0_ = 0.01, *p*_0_ = .75; *ω*_1_ = 1.00, *p*_1_ = .02; *ω*_2_ = 0.99, *p*_2_ = .23	.13
	M2a‐rel	111	−19,640.12	*ω*_0_ = 0.01, *p*_0_ = .75; *ω*_1_ = 1.00, *p*_1_ = .02; *ω*_2_ = 0.18, *p*_2_ = .23	

## 4 DISCUSSION

In this study, we tested for climatic correlates of mitochondrial DNA variation in lacertid lizards. We constructed phylogenetic relationships within Lacertidae, which were generally consistent with the pattern reported by Pyron et al. (2013). Our study is the first to demonstrate that three mitochondrial genes (*ATP6*, *ATP8*, and *ND3*) are under positive selection by climate in lacertid lizards. More specifically, two temperature‐related (Bio3 and Bio4) and two precipitation‐related (Bio13 and Bio15) climatic variables contributed significantly to variation in these three positive selection genes.

The gene order in the mitogenome always varies in invertebrates (Pereira, 2000; Yamazaki et al., 1997; Zhang et al., 2021). Gene reversal, gene transposition, and tandem duplications with subsequent random gene loss have been proved to be possible causes of changes in the mitochondrial gene order in deep‐sea mussels (Zhang et al., 2021). In contrast, the mitochondrial gene order is conserved in vertebrates (Kundu et al., 2018; Tian & Guo, 2022). Our study supports the conclusion drawn in vertebrates, as revealed by the observation that the mitochondrial gene order was almost identical among different species of lacertid lizards, with inter‐specific differences reflected only in whether some genes are located on the heavy or light strand. The AT and GC skews are a measure of strand asymmetry, and relate to gene replication, selection, and mutation; the GC skews are always higher than the AT skews (Hassanin, 2006; Wei et al., 2010). Our data confirmed that the GC skews were higher than the AT skews among lacertid species. In addition, the similar GC skews among lacertid species may reflect the similar strand asymmetry in these reptiles. Strand bias is usually higher in Arthropodas than in reptiles (Hassanin, 2006; Kundu et al., 2018; Wei et al., 2010), indicating that the frequency of gene mutation may be higher in invertebrates than in vertebrates.

Environmental stressors may result in gene mutation. Each population will produce selection signatures to adapt to its indigenous environments, and then the evolution of genes occurs (Fleming et al., 2017; Pedro et al., 2015). Genetic differences are one of the most important indicators for testing species evolutionary relationships. In this study, the phylogenetic relationship of the Lacertidae family based on 15 mitochondrial genes is basically consistent with previous studies, which ensured the accuracy of subsequent research (Arnold et al., 2007; Pyron et al., 2013). Phylogenetic relationships inferred from mtDNA data show a strong correlation with the climate, indicating that the mitochondrial genes are affected by climate change. In agreement with previous studies on a diverse array of animal taxa including humans (Balloux et al., 2009; Lamb et al., 2018; Mishmar et al., 2003; Sun et al., 2018; Zhang et al., 2013), mitochondrial genes can be selected by climate in lizards, as revealed by the observation that three mitochondrial genes (*ATP6*, *ATP8*, and *ND3*) were under positive selection by climate in lacertid lizards. ATP6 is the mtDNA‐encoded subunit that is incorporated into OXPHOS complex V, which has a channel for the protons' flow back into the mitochondrial matrix (da Fonseca et al., 2008; Fernandez‐Vizarra et al., 2009; Wittig et al., 2010). ATP8 is a companion protein of ATP6 in the ATP synthase (Mishmar et al., 2003; Neckelmann et al., 1987; Wallace et al., 1987). ND3 is oxidoreductase, also known as subunits of complex I (NADH dehydrogenase) (Mishmar et al., 2003), which plays essential role in cellular energy production and proton transport (Efremov et al., 2010; Ohnishi, 2010). The gene mutation of *ATP* and *ND* will affect the efficiency of proton translocation, and balance the generate heat and ATP synthesis (Brand, 2000; Sun et al., 2018; Wallace, 2005). Therefore, the gene selection of *ATP* and *ND* is simultaneous (Slimen et al., 2017; Sun et al., 2018). Similarly, the climate promotes the gene selection of *ATP6*, *ATP8*, and *ND3* and increases the adaption to different climate in lizards.

Temperature‐ and precipitation‐related climatic variables are more often equally represented in genetic variation and gene flow in plants (Postolache et al., 2021; Wang et al., 2020). In animals, however, temperature‐related climatic variables always attract more attention (Ballard et al., 2007; Efremov et al., 2010; Elson et al., 2004). In this study, we simultaneously paid attention to temperature‐ and precipitation‐related factors. In agreement with earlier studies on two species of hares (*Lepus capensis*, Slimen et al., 2017; *Lepus europaeus*, Stefanovic et al., 2019), we found that the precipitation factors were the drivers of positive selection of mitochondrial genes in lacertid lizards. Bio3, Bio4, and Bio13 are the common bioclimatic variables affecting the distribution of animals and ecologically suitable niches (Deng et al., 2022; Gao et al., 2021; Moradi‐Asl et al., 2020; Ye et al., 2022). According to our research, these bioclimatic variables were mostly important for gene selection, and increased the adaptability to different environment of lizards.

## 5 CONCLUSIONS

Climate‐driven mitochondrial selection remains sparsely studied in reptiles. Here, we reported the complete mitochondrial genome sequence of a lacertid lizard (*T. intermedius*) and used mitogenomes from 54 lacertid species including *T. intermedius* to study their phylogenetic relationships and to identify the mitochondrial genes under positive selection by climate. We found that the length (17,713 bp) of the complete mitochondrial genome sequence of *T. intermedius* was within the range of lengths (17,224–18,943) already reported for other congeneric species. The arrangement of mitochondrial genes in *T. intermedius* was the same as in other congeneric species, providing an additional support for the idea that the mitochondrial gene order is conserved in vertebrates. The environmental association of mitochondrial selection is evident, as revealed by the observation that the 54 lacertid species could be divided into three geographically and climatically different clades. We identified three mitochondrial genes (*ATP*6, *ATP8*, and *ND3*) under positive selection by climate, and found that isothermality, temperature seasonality, precipitation of wettest month, and precipitation seasonality were the most important climatic variables contributing to the gene selection.

## AUTHOR CONTRIBUTIONS

**Xiang Zhang:** Data curation (equal). **Jian Chen:** Data curation (equal). **Hong‐Yu Luo:** Data curation (supporting). **Xin Chen:** Data curation (supporting). **Jun Zhong:** Formal analysis (equal); writing – original draft (lead); writing – review and editing (lead). **Xiang Ji:** Writing – review and editing (supporting).

## CONFLICT OF INTEREST STATEMENT

The authors have no conflicts of interest to declare.

### OPEN RESEARCH BADGES

Environmental data can be obtained from WorldClim (http://www.worldclim.org). The other informations are within the article and its supplementary material.

## Supporting information


Table S1.

Table S2.

Table S3.

Table S4.

## Data Availability

The Complete mitochondrial genomes of *T. intermedius* was uploaded to NCBI (GenBank accession Number OQ632596). The primer information and mitochondrial genomes from NCBI are within the supplementary material.

## References

[ece311176-bib-0001] Arnold, E. N. (1997). Interrelationships and evolution of the east Asian grass lizards, *Takydromus* (Squamata: Lacertidae). Zoolological Journal of the Linnean Society, 119, 267–296. 10.1006/zjls.1996.0067

[ece311176-bib-0002] Arnold, E. N. , Arribas, O. , & Carranza, S. (2007). Systematics of the Palaearctic and oriental lizard tribe Lacertini (Squamata: Lacertidae: Lacertinae), with descriptions of eight new genera. Zootaxa, 1430, 1–86. 10.11646/zootaxa.1430.1.1

[ece311176-bib-0003] Ballard, J. W. O. , Melvin, R. G. , Katewa, S. D. , & Maas, K. (2007). Mitochondrial DNA variation is associated with measurable differences in life‐history traits and mitochondrial metabolism in *Drosophila simulans* . Evolution, 61, 1735–1747. 10.1111/j.1558-5646.2007.00133.x 17598752

[ece311176-bib-0004] Ballard, J. W. O. , & Pichaud, N. (2014). Mitochondrial DNA: More than an evolutionary bystander. Functional Ecology, 28, 218–231. 10.1111/1365-2435.12177

[ece311176-bib-0005] Balloux, F. , Handley, L. J. L. , Jombart, T. , Liu, H. , & Manica, A. (2009). Climate shaped the worldwide distribution of human mitochondrial DNA sequence variation. Proceedings of the Royal Society B, 276, 3447–3455. 10.1098/rspb.2009.0752 19586946 PMC2817182

[ece311176-bib-0006] Boore, J. L. (1999). Animal mitochondrial genomes. Nucleic Acids Research, 27, 1767–1780. 10.1093/nar/27.8.1767 10101183 PMC148383

[ece311176-bib-0007] Brand, M. D. (2000). Uncoupling to survive? The role of mitochondrial inefficiency in ageing. Experimental Gerontology, 35, 811–820. 10.1016/S0531-5565(00)00135-2 11053672

[ece311176-bib-0008] Chen, Q.‐L. , Tang, X.‐S. , Yao, W.‐J. , & Lu, S.‐Q. (2009). Bioinformatics analysis the complete sequences of cytochrome *b* of *Takydromus sylvaticus* and modeling the tertiary structure of encoded protein. International Journal of Biological Sciences, 5, 596–602. 10.3724/SP.J.1231.2010.06703 19774111 PMC2748471

[ece311176-bib-0009] Chong, R. A. , & Mueller, R. L. (2013). Low metabolic rates in salamanders are correlated with weak selective constraints on mitochondrial genes. Evolution, 67, 894–899. 10.1111/j.1558-5646.2012.01830.x 23461338

[ece311176-bib-0010] da Fonseca, R. R. , Johnson, W. E. , O'Brien, S. J. , Ramos, M. J. , & Antunes, A. (2008). The adaptive evolution of the mammalian mitochondrial genome. BMC Genomics, 9, 119. 10.1186/1471-2164-9-119 18318906 PMC2375446

[ece311176-bib-0011] Deng, X.‐Q. , Xu, D.‐P. , Liao, W.‐K. , Wang, R.‐L. , & Zhuo, Z.‐H. (2022). Predicting the distributions of *Scleroderma guani* (Hymenoptera: Bethylidae) under climate change in China. Ecology and Evolution, 12, e9410. 10.1002/ece3.9410 36225826 PMC9534726

[ece311176-bib-0012] Dupoue, A. , Brischoux, F. , & Lourdais, O. (2017). Climate and foraging mode explain interspecific variation in snake metabolic rates. Proceedings of the Royal Society B, 284, 20172108. 10.1098/rspb.2017.2108 29142118 PMC5719182

[ece311176-bib-0013] Efremov, R. G. , Baradaran, R. , & Sazanov, L. A. (2010). The architecture of respiratory complex I. Nature, 465, 441–445. 10.1038/nature09066 20505720

[ece311176-bib-0014] Ellis, N. , Smith, S. J. , & Pitcher, C. R. (2012). Gradient forests: Calculating importance gradients on physical predictors. Ecology, 93, 156–168. 10.1890/11-0252.1 22486096

[ece311176-bib-0015] Elson, J. L. , Turnbull, D. M. , & Howell, N. (2004). Comparative genomics and the evolution of human mitochondrial DNA: Assessing the effects of selection. Americn Journal of Human Genetics, 74, 229–238. 10.1086/381505 PMC1181921 14712420

[ece311176-bib-0016] Fernandez‐Vizarra, E. , Tiranti, V. , & Zeviani, M. (2009). Assembly of the oxidative phosphorylation system in humans: What we have learned by studying its defects. Biochimica et Biophysica Acta‐Molecular Cell Research, 1793, 200–211. 10.1016/j.bbamcr.2008.05.028 18620006

[ece311176-bib-0017] Fleming, D. S. , Weigend, S. , Simianer, H. , Weigend, A. , Rothschild, M. , Schmidt, C. , Ashwell, C. , Persia, M. , Reecy, J. , & Lamont, S. J. (2017). Genomic comparison of indigenous African and northern European chickens reveals putative mechanisms of stress tolerance related to environmental selection pressure. G3: Genes, Genomes, Genetics, 7, 1525–1537. 10.1534/g3.117.041228 28341699 PMC5427493

[ece311176-bib-0018] Forsberg, R. , & Christiansen, F. B. (2003). A codon‐based specific selection in parasites, with an application influenza a virus. Molecular Biology and Evolution, 20, 1252–1259. 10.1093/molbev/msg149 12777510

[ece311176-bib-0019] Franks, S. J. , & Hoffmann, A. A. (2012). Genetics of climate change adaptation. Annual Review of Genetics, 46, 185–208. 10.1146/annurev-genet-110711-155511 22934640

[ece311176-bib-0020] Gao, F.‐L. , Chen, C.‐J. , Arab, D. A. , Du, Z.‐G. , He, Y.‐H. , & Ho, S. Y. W. (2019). EasyCodeML: A visual tool for analysis of selection using CodeML. Ecology and Evolution, 9, 3891–3898. 10.1002/ece3.5015 31015974 PMC6467853

[ece311176-bib-0021] Gao, T. , Xu, Q. , Liu, Y. , Zhao, J.‐Q. , & Shi, J. (2021). Predicting the potential geographic distribution of sirex nitobei in China under climate change using maximum entropy model. Forests, 12, 151. 10.3390/f12020151

[ece311176-bib-0022] Guo, K. , Zhong, J. , Xie, F. , Zhu, L. , Qu, Y.‐F. , & Ji, X. (2021). Climate warming will increase chances of hybridization and introgression between two *Takydromus* lizards (Lacertidae). Ecology and Evolution, 11, 8573–8584. 10.1002/ece3.7671 34257917 PMC8258214

[ece311176-bib-0023] Guo, K. , Zhong, J. , Zhu, L. , Xie, F. , Du, Y. , & Ji, X. (2021). The thermal dependence and molecular basis of physiological color change in *Takydromus septentrionalis* (Lacertidae). Biology Open, 10, bio058503. 10.1242/bio.058503 33593793 PMC8015239

[ece311176-bib-0024] Guo, X.‐G. , Dai, X. , Chen, D.‐L. , Papenfuss, T. J. , Ananjeva, N. B. , Melnikov, D. A. , & Wang, Y.‐Z. (2011). Phylogeny and divergence times of some racerunner lizards (Lacertidae: *Eremias*) inferred from mitochondrial 16S rRNA gene segments. Molecular Phylogenetics and Evolution, 61, 400–412. 10.1016/j.ympev.2011.06.022 21767655

[ece311176-bib-0025] Hassanin, A. (2006). Phylogeny of Arthropoda inferred from mitochondrial sequences: Strategies for limiting the misleading effects of multiple changes in pattern and rates of substitution. Molecular Phylogenetics and Evolution, 38, 100–116. 10.1016/j.ympev.2005.09.012 16290034

[ece311176-bib-0026] He, J.‐Z. , Xiu, M.‐H. , Tang, X.‐L. , Yue, F. , Wang, N.‐B. , Yang, S.‐B. , & Chen, Q. (2013). The different mechanisms of hypoxic acclimatization and adaptation in lizard *Phrynocephalus vlangalii* living on Qinghai‐Tibet plateau. Journal of Experimental Zoology A, 319, 117–123. 10.1002/jez.1776 23319459

[ece311176-bib-0027] Ji, X. , Du, W.‐G. , Lin, L.‐H. , & Luo, L.‐G. (2007). Measuring temporal variation in reproductive output reveals optimal resource allocation to reproduction in the northern grass lizard, *Takydromus septentrionalis* . Biological Journal of the Linnean Society, 91, 315–324. 10.1111/j.1095-8312.2007.00791.x

[ece311176-bib-0028] Kimura, M. (1979). The neutral theory of molecular evolution. Scientific American, 241, 98–108. 10.1038/scientificamerican1179-98 504979

[ece311176-bib-0029] Kovac, H. , Kundegraber, B. , Kaefer, H. , Petrocelli, I. , & Stabentheiner, A. (2020). Relation between activity, endothermic performance and respiratory metabolism in two paper wasps: *Polistes dominula* and *Polistes gallicus* . Comparative Biochemistry and Physiology A, 250, 110804. 10.1016/j.cbpa.2020.110804 32920209

[ece311176-bib-0030] Kumar, S. , Stecher, G. , & Tamura, K. (2016). MEGA7: Molecular evolutionary genetics analysis version 7.0 for bigger datasets. Molecular Biology and Evolution, 33, 1870–1874. 10.1093/molbev/msw054 27004904 PMC8210823

[ece311176-bib-0031] Kundu, S. , Kumar, V. , Tyagi, K. , Chakraborty, R. , Singha, D. , Rahaman, I. , Pakrashi, A. , & Chandra, K. (2018). Complete mitochondrial genome of black soft‐shell turtle (*Nilssonia nigricans*) and comparative analysis with other Trionychidae. Scientific Reports, 8, 17378. 10.1038/s41598-018-35822-5 30478342 PMC6255766

[ece311176-bib-0032] Lamb, A. M. , Gan, H. M. , Greening, C. , Joseph, L. , Lee, Y. P. , Moran‐Ordonez, A. , Sunnucks, P. , & Pavlova, A. (2018). Climate‐driven mitochondrial selection: A test in Australian songbirds. Molecular Ecology, 27, 898–918. 10.1111/mec.14488 29334409

[ece311176-bib-0033] Lin, Z.‐H. , & Ji, X. (2005). Partial tail loss has no severe effects on energy stores and locomotor performance in a lacertid lizard, *Takydromus septentrionalis* . Journal of Comparative Physiology B, 175, 567–573. 10.1007/s00360-005-0017-z 16133493

[ece311176-bib-0034] Luo, L.‐G. , Ding, G.‐H. , & Ji, X. (2010). Income breeding and temperature‐induced plasticity in reproductive traits in lizards. Journal of Experimental Biology, 213, 2073–2078. 10.1242/jeb.041137 20511521

[ece311176-bib-0035] Luo, Y.‐J. , Gao, W.‐X. , Gao, Y.‐Q. , Tang, S. , Huang, Q.‐Y. , Tan, X.‐L. , Chen, J. , & Huang, T.‐S. (2008). Mitochondrial genome analysis of *Ochotona curzoniae* and implication of cytochrome *c* oxidase in hypoxic adaptation. Mitochondrion, 8, 352–357. 10.1016/j.mito.2008.07.005 18722554

[ece311176-bib-0036] Ma, L. , Liu, P. , Su, S. , Luo, L.‐G. , Zhao, W.‐G. , & Ji, X. (2019). Life‐history consequences of local adaptation in lizards: *Takydromus wolteri* (Lacertidae) as a model organism. Biological Journal of the Linnean Society, 127, 88–99. 10.1093/biolinnean/blz024

[ece311176-bib-0037] Mishmar, D. , Ruiz‐Pesini, E. , Golik, P. , Macaulay, V. , Clark, A. G. , Hosseini, S. , Brandon, M. , Easley, K. , Chen, E. , Brown, M. D. , Sukernik, R. I. , Olckers, A. , & Wallace, D. C. (2003). Natural selection shaped regional mtDNA variation in humans. Proceedings of the National Academy of Sciences of the United States of America, 100, 171–176. 10.1073/pnas.0136972100 12509511 PMC140917

[ece311176-bib-0038] Moradi‐Asl, E. , Mohebali, M. , Rassi, Y. , Vatandoost, H. , & Saghafipour, A. (2020). Environmental variables associated with distribution of canine visceral leishmaniasis in dogs in ardabil province, northwestern Iran: A systematic review. Iranian Journal of Public Health, 49, 1033–1044. 10.18502/ijph.v49i6.3354

[ece311176-bib-0039] Neckelmann, N. , Li, K. , Wade, R. P. , Shuster, R. , & Wallace, D. C. (1987). cDNA sequence of a human skeletal muscle ADP/ATP translocator: Lack of a leader peptide, divergence from a fibroblast translocator cDNA, and coevolution with mitochondrial DNA genes. Proceedings of the National Academy of Sciences of the United States of America, 84, 7580–7584. 10.1073/pnas.84.21.7580 2823266 PMC299343

[ece311176-bib-0040] Ohnishi, T. (2010). Structural biology: Piston drives a proton pump. Nature, 465, 428–429. 10.1038/465428a 20505714

[ece311176-bib-0041] Olson, M. S. , Levsen, N. , Soolanayakanahally, R. Y. , Guy, R. D. , Schroeder, W. R. , Keller, S. R. , & Tiffin, P. (2013). The adaptive potential of *Populus balsamifera* L. to phenology requirements in a warmer global climate. Molecular Ecology, 22, 1214–1230. 10.1111/mec.12067 23094714

[ece311176-bib-0042] Orme, D. , Freckleton, R. , Thomas, G. , Petzoldt, T. , Fritz, S. , Isaac, N. , & Pearse, W. (2018). The Caper package: Comparative analysis of phylogenetics and evolution in R . https://cran.r‐project.org/web/packages/caper/index.html

[ece311176-bib-0043] Pedro, S. S. L. , Alves, J. M. P. , Barreto, A. S. , & Lima, A. O. D. (2015). Evidence of positive selection of aquaporins genes from *Pontoporia blainvillei* during the evolutionary process of cetaceans. PLoS One, 10, e0134516. 10.1371/journal.pone.0134516 26226365 PMC4520692

[ece311176-bib-0044] Pereira, S. L. (2000). Mitochondrial genome organization and vertebrate phylogenetics. Genetics and Molecular Biology, 23, 745–752. 10.1590/S1415-47572000000400008

[ece311176-bib-0045] Pichaud, N. , Ekstrom, A. , Breton, S. , Sundstrom, F. , Rowinski, P. , Blier, P. U. , & Sandblom, E. (2020). Adjustments of cardiac mitochondrial phenotype in a warmer thermal habitat is associated with oxidative stress in European perch, *Perca fluviatilis* . Scientific Reports, 10, 17697. 10.1038/s41598-020-74788-1 33077851 PMC7572411

[ece311176-bib-0046] Postolache, D. , Oddou‐Muratorio, S. , Vajana, E. , Bagnoli, F. , Guichoux, E. , Hampe, A. , Le Provost, G. , Lesur, I. , Popescu, F. , Scotti, I. , Piotti, A. , & Vendramin, G. G. (2021). Genetic signatures of divergent selection in European beech (*Fagus sylvatica* L.) are associated with the variation in temperature and precipitation across its distribution range. Molecular Ecology, 30, 5029–5047. 10.1111/mec.16115 34383353

[ece311176-bib-0500] Pyron, R. A. , Burbrink, F. T. , & Wiens, J. J. (2013). A phylogeny and revised classification of squamata, including 4161 species of lizards and snakes. BMC Evolutionary Biology, 13, 93. 10.1186/1471-2148-13-93 23627680 PMC3682911

[ece311176-bib-0047] Ronquist, F. , Teslenko, M. , van der Mark, P. , Ayres, D. L. , Darling, A. , Höhna, S. , Larget, B. , Liu, L. , Suchard, M. A. , & Huelsenbeck, J. P. (2012). MrBayes 3.2: Efficient Bayesian phylogenetic inference and model choice across a large model space. Systematic Biology, 61, 539–542. 10.1093/sysbio/sys029 22357727 PMC3329765

[ece311176-bib-0048] Scott, G. R. , Schulte, P. M. , Egginton, S. , Scott, A. L. M. , Richards, J. G. , & Milsom, W. K. (2011). Molecular evolution of cytochrome c oxidase underlies high‐altitude adaptation in the bar‐headed goose. Molecular Biology and Evolution, 28, 351–363. 10.1093/molbev/msq205 20685719

[ece311176-bib-0049] Sikdar, M. (2023). Complete mitochondrial DNA sequence tries to settle hitherto putative history of Kayastha population of India. American Journal of Human Biology, 35, e23851. 10.1002/ajhb.23851 36571462

[ece311176-bib-0050] Slimen, H. B. , Schaschl, H. , Knauer, F. , & Suchentrunk, F. (2017). Selection on the mitochondrial *ATP* s*ynthase 6* and the *NADH dehydrogenase 2* genes in hares (*Lepus capensis* L.,1758) from a steep ecological gradient in North Africa. BMC Evolutionary Biology, 17, 46. 10.1186/s12862-017-0896-0 28173765 PMC5297179

[ece311176-bib-0051] Stefanovic, M. , Djan, M. , Velickovic, N. , Beukovic, D. , Lavadinovic, V. , Zhelev, C. D. , Demirbas, Y. , Paule, L. , Gedeon, C. I. , Mamuris, Z. , Posautz, A. , Beiglbock, C. , Kuebber‐Heiss, A. , & Suchentrunk, F. (2019). Positive selection and precipitation effects on the mitochondrial *NADH* dehydrogenase subunit 6 gene in brown hares (*Lepus europaeus*) under a phylogeographic perspective. PLoS One, 14, e0224902. 10.1371/journal.pone.0224902 31703111 PMC6839855

[ece311176-bib-0052] Sun, J.‐T. , Jin, P.‐Y. , Hoffmann, A. A. , Duan, X.‐Z. , Dai, J. , Hu, G. , Xue, X.‐F. , & Hong, X.‐Y. (2018). Evolutionary divergence of mitochondrial genomes in two *Tetranychus* species distributed across different climates. Insect Molecular Biology, 27, 698–709. 10.1111/imb.12501 29797479

[ece311176-bib-0053] Tian, L.‐L. , & Guo, X.‐G. (2022). Complete mitochondrial genomes of five racerunners (Lacertidae: *Eremias*) and comparison with other lacertids: Insights into the structure and evolution of the control region. Genes, 13, 726. 10.3390/genes13050726 35627111 PMC9141765

[ece311176-bib-0054] Volis, S. , Zhang, Y.‐H. , Deng, T. , Dorman, M. , Blecher, M. , & Abbott, R. J. (2019). Divergence and reproductive isolation between two closely related allopatric *Iris* species. Biological Journal of the Linnean Society, 127, 377–389. 10.1093/biolinnean/blz014

[ece311176-bib-0055] Wallace, D. C. (2005). A mitochondrial paradigm of metabolic and degenerative diseases, aging, and cancer: A dawn for evolutionary medicine. Annual Review of Genetics, 39, 359–407. 10.1146/annurev.genet.39.110304.095751 PMC2821041 16285865

[ece311176-bib-0056] Wallace, D. C. , Ye, J.‐H. , Neckelmann, S. N. , Singh, G. , Webster, K. A. , & Greenberg, B. D. (1987). Sequence analysis of cDNAs for the human and bovine *ATP* synthase beta subunit: Mitochondrial DNA genes sustain seventeen times more mutations. Current Genetics, 12, 81–90. 10.1007/BF00434661 2896550

[ece311176-bib-0057] Wang, J.‐N. , Hu, Z.‐B. , Upadhyaya, H. D. , & Morris, G. P. (2020). Genomic signatures of seed mass adaptation to global precipitation gradients in sorghum. Heredity, 124, 108–121. 10.1038/s41437-019-0249-4 31316156 PMC6906510

[ece311176-bib-0058] Weadick, C. J. , & Chang, B. S. W. (2012). An improved likelihood ratio test for detecting site‐specific functional divergence among clades of protein‐coding genes. Molecular Biology and Evolution, 29, 1297–1300. 10.1093/molbev/msr311 22319160

[ece311176-bib-0059] Wei, S.‐J. , Shi, M. , Chen, X.‐X. , Sharkey, M. J. , van Achterberg, C. , Ye, G.‐Y. , & He, J.‐H. (2010). New views on strand asymmetry in insect mitochondrial genomes. PLoS One, 5, e12708. 10.1371/journal.pone.0012708 20856815 PMC2939890

[ece311176-bib-0060] White, C. R. , Alton, L. A. , & Frappell, P. B. (2012). Metabolic cold adaptation in fishes occurs at the level of whole animal, mitochondria and enzyme. Proceedings of the Royal Society B, 279, 1740–1747. 10.1098/rspb.2011.2060 22158960 PMC3297453

[ece311176-bib-0061] Wittig, I. , Meyer, B. , Heide, H. , Steger, M. , Bleier, L. , Wumaier, Z. , Karas, M. , & Schagger, H. (2010). Assembly and oligomerization of human ATP synthase lacking mitochondrial subunits a and A6L. Biochimica et Biophysica Acta‐Bioenergetics, 1797, 1004–1011. 10.1016/j.bbabio.2010.02.021 20188060

[ece311176-bib-0062] Xia, T. , Zhang, H.‐H. , Zhang, L. , Yang, X.‐F. , Sun, G.‐L. , Chen, J. , Xu, D.‐J. , & Zhao, C. (2019). Comparative and evolutionary analysis of the reptilian hedgehog gene family (*Shh*, *Dhh*, and *Ihh*). PeerJ, 7, e7613. 10.7717/peerj.7613 31531274 PMC6718155

[ece311176-bib-0063] Xu, S.‐Q. , Yang, Y.‐Z. , Zhou, J. , Jing, G.‐E. , Chen, Y.‐T. , Wang, J. , Yang, H.‐M. , Wang, J. , Yu, J. , Zheng, X.‐G. , & Ge, R.‐L. (2005). A mitochondrial genome sequence of the Tibetan antelope (*Pantholops hodgsonii*). Genomics, Proteomics & Bioinformatics, 3, 5–17. 10.1016/S1672-0229(05)03003-2 PMC5172476 16144518

[ece311176-bib-0064] Yamazaki, N. , Ueshima, R. , Terrett, J. A. , Yokobori, S. I. , Kaifu, M. , Segawa, R. , Kobayashi, T. , Numachi, K. I. , Ueda, T. , Nishikawa, K. , Watanabe, K. , & Thomas, R. H. (1997). Evolution of pulmonate gastropod mitochondrial genomes: Comparisons of gene organizations of *Euhadra*, *Cepaea* and *Albinaria* and implications of unusual tRNA secondary structures. Genetics, 145, 749–758. 10.1093/genetics/145.3.749 9055084 PMC1207859

[ece311176-bib-0065] Yang, Z.‐H. (2007). PAML 4: Phylogenetic analysis by maximum likelihood. Molecular Biology and Evolution, 24, 1586–1591. 10.1093/molbev/msm088 17483113

[ece311176-bib-0066] Ye, X.‐L. , Wu, Q. , Li, X.‐R. , & Zhao, X.‐M. (2022). Incorporating interspecific relationships into species distribution models can better assess the response of species to climate change, a case study of two Chinese primates. Ecological Indictors, 142, 109255. 10.1016/j.ecolind.2022.109255

[ece311176-bib-0067] Zhang, H.‐X. , Luo, Q.‐B. , Sun, J. , Liu, F. , Wu, G. , Yu, J. , & Wang, W.‐W. (2013). Mitochondrial genome sequences of *Artemia tibetiana* and *Artemia urmiana*: Assessing molecular changes for high plateau adaptation. Science China. Life Sciences, 56, 440–452. 10.1007/s11427-013-4474-4 23633076

[ece311176-bib-0068] Zhang, K. , Sun, J. , Xu, T. , Qiu, J.‐W. , & Qian, P.‐Y. (2021). Phylogenetic relationships and adaptation in deep‐sea mussels: Insights from mitochondrial genomes. International Journal of Molecular Sciences, 22, 1900. 10.3390/ijms22041900 33672964 PMC7918742

