# Supplementary material for: Climate‐driven mitochondrial selection in lacertid lizards
Source: Ecol Evol. 2024 Mar 24;14(3):e11176. doi: 10.1002/ece3.11176 (PMC10961475; doi:10.1002/ece3.11176)
Supplement: Supplementary file 1 — Table S1. [file ECE3-14-e11176-s004.doc]

Table S1 The primers used in analysis of the *T. intermedius* mitochondrial genome.

| Code | Primer sequence (5`-3`) | Approximate product length (bp) | Approximate Tm (°C) |
| --- | --- | --- | --- |
| 1 | F: GTCATTGTAGCTTATTTTTGAA  R: TGTCTTTGCTTTTTTATGCTTA | 1200 | 50 |
| 2 | F: TAGCTTATTAAAGCACCTAGC  R: ACTCTTGAAGGGGATGGCGCTG | 1400 | 55 |
| 3 | F: TTTAGTTGGGGCGACTTCGGA  R: GATATTTATGTATCTAGCTAGG | 1200 | 54 |
| 4 | F: TGAGCATCCAACTCAAAATATGC  R: AAGTGTTTGAGTTGCATTCAGA | 1200 | 53 |
| 5 | F: CAATAAAATGACGATTTAAAC  R: GCCAAATGGTTCTTTTTTACCA | 1200 | 58 |
| 6 | F: CAACCTGTATTAACATAAAACC  R: CTGTATATTCGTAGCTTCAGTA | 1600 | 50 |
| 7 | F: ATAGATGCCCAGGAGGTAGAGA  R: CTCGTCATCATTGTTGTATAGT | 1500 | 56 |
| 8 | F: TAATGACCCACCAAACACAC  R: GTCAGAATTTATTAAGTCGAAA | 1200 | 52 |
| 9 | F: CCAACCCCTTGAGCAATTAAC  R: CTAACCCCATGTGGCTTACAGA | 1300 | 55 |
| 10 | F: AAACTCGGAGGATATGGTCTCATTC  R: TTGATGTGAAGAGCAATATTTTGTCC | 2400 | 55 |
| 11 | F: ACACAAAATGATATTAAAAAGATTATTGCC  R: GGTTGTTTGAGCCTGTTTCATG | 2000 | 54 |
| 12 | F: TAATGGCACACCACTTAAATCC  R: TTGGTTTACAAGACCAATGCTT | 1600 | 52 |
| 13 | F: TATACCATTAACCCATTTATCAAAACAA  R: GGGGTAAATATATTAATGCATAATTAAA | 1400 | 54 |
| 14 | F: GACACAACCAAATCATCTTCTACACA  R: GTTGACTGCAGTTAACCGATAATTGA | 1300 | 55 |
| 15 | F: AACAAAATATGTTACCTTTGTC  R: TATTACTGCTGATCACCCGTGG | 600 | 55 |
