# Supplementary material for: Climate‐driven mitochondrial selection in lacertid lizards
Source: Ecol Evol. 2024 Mar 24;14(3):e11176. doi: 10.1002/ece3.11176 (PMC10961475; doi:10.1002/ece3.11176)
Supplement: Supplementary file 2 — Table S2. [file ECE3-14-e11176-s003.doc]

Table S2 Complete mitochondrial genomes of 56 (54 lacertid and 2 gekkonid) species of lizards used in this study.

|  | Species | Length (bp) | Accession NO. | Reference |
| --- | --- | --- | --- | --- |
| 1 | *Acanthodactylus aureus* | 17021 | NC_059775 | Kirchhof et al., 2021 |
| 2 | *Acanthodactylus boskianus* | 17143 | NC_059772 | Kirchhof et al., 2021 |
| 3 | *Acanthodactylus erythrurus* | 16827 | NC_059773 | Kirchhof et al., 2021 |
| 4 | *Acanthodactylus guineensis* | 16963 | NC_059781 | Kirchhof et al., 2021 |
| 5 | *Acanthodactylus schmidti* | 16943 | NC_059782 | Kirchhof et al., 2021 |
| 6 | *Algyroides nigropunctatus* | 15844 | NC_059780 | Kirchhof et al., 2021 |
| 7 | *Australolacerta australis* | 17019 | NC_059777 | Kirchhof et al., 2021 |
| 8 | *Darevskia armeniaca* | 17521 | MG704915 | Murtskhvaladze et al., 2020 |
| 9 | *Darevskia brauneri* | 16976 | MH481137 | Murtskhvaladze et al., 2020 |
| 10 | *Darevskia caucasica* | 16343 | MH481131 | Murtskhvaladze et al., 2020 |
| 11 | *Darevskia chlorogaster* | 17479 | MH481136 | Murtskhvaladze et al., 2020 |
| 12 | *Darevskia clarkorum* | 16301 | MH481134 | Murtskhvaladze et al., 2020 |
| 13 | *Darevskia daghestanica* | 17189 | MH481135 | Murtskhvaladze et al., 2020 |
| 14 | *Darevskia dahli* | 17528 | MG704916 | Murtskhvaladze et al., 2020 |
| 15 | *Darevskia derjugini* | 16960 | MH481130 | Murtskhvaladze et al., 2020 |
| 16 | *Darevskia mixta* | 17532 | MG704917 | Murtskhvaladze et al., 2020 |
| 17 | *Darevskia parvula* | 17510 | MG704918 | Murtskhvaladze et al., 2020 |
| 18 | *Darevskia portschinskii* | 17529 | MG704919 | Murtskhvaladze et al., 2020 |
| 19 | *Darevskia praticola* | 16418 | MH481132 | Murtskhvaladze et al., 2020 |
| 20 | *Darevskia raddei* | 20478 | MH481133 | Murtskhvaladze et al., 2020 |
| 21 | *Darevskia rudis* | 17534 | MG704920 | Murtskhvaladze et al., 2020 |
| 22 | *Darevskia unisexualis* | 21433 | KX644918 | Komissarov et al., 2016 |
| 23 | *Darevskia valentini* | 17393 | NC_045934 | Murtskhvaladze et al., 2020 |
| 24 | *Eremias argus* | 18521 | JQ086345 | Unpublished |
| 25 | *Eremias brenchleyi* | 19542 | EF490071 | Unpublished |
| 26 | *Eremias dzungarica* | 19899 | MW250881 | Wang et al., 2021 |
| 27 | *Eremias multiocellata* | 18996 | KJ664798 | Unpublished |
| 28 | *Eremias nikolskii* | 20840 | NC_060561 | Unpublished |
| 29 | *Eremias przewalskii* | 18225 | NC_025929 | Du et al., 2014 |
| 30 | *Eremias scripta* | 19381 | OM935766 | Tian et al., 2022 |
| 31 | *Eremias stummeri* | 19602 | NC_029878 | Zhou et al., 2015 |
| 32 | *Eremias szczerbaki* | 19650 | NC_062143 | Unpublished |
| 33 | *Eremias vermiculata* | 19796 | KP981388 | Unpublished |
| 34 | *Eremias yarkandensis* | 18743 | NC_060637 | Unpublished |
| 35 | *Gallotia atlantica* | 15552 | NC_059771 | Kirchhof et al., 2021 |
| 36 | *Lacerta agilis* | 19093 | CM020436 | Unpublished |
| 37 | *Lacerta bilineata* | 17154 | KT722705 | Unpublished |
| 38 | *Lacerta viridis* | 17156 | NC_008328 | Böhme et al., 2007 |
| 39 | *Meroles squamulosus* | 16860 | NC_059779 | Kirchhof et al., 2021 |
| 40 | *Mesalina olivieri* | 16899 | NC_059774 | Kirchhof et al., 2021 |
| 41 | *Pedioplanis laticeps* | 17046 | NC_059778 | Kirchhof et al., 2021 |
| 42 | *Phoenicolacerta kulzeri* | 17199 | FJ460596 | Podnar et al., 2009 |
| 43 | *Podarcis muralis* | 17311 | NC_011607 | Podnar et al., 2009 |
| 44 | *Podarcis siculus* | 17286 | MH157278 | Buglione et al., 2019 |
| 45 | *Psammodromus algirus* | 17118 | NC_059776 | Kirchhof et al., 2021 |
| 46 | *Takydromus amurensis* | 17333 | NC_030209 | Ma et al., 2016 |
| 47 | *Takydromus intermedius* | 17713 | OQ632596 | This study |
| 48 | *Takydromus kuehnei* | 17224 | MZ435950 | Wu et al., 2022 |
| 49 | *Takydromus septentrionalis* | 18304 | MK630237 | Hu et al., 2019 |
| 50 | *Takydromus sexlineatus* | 18943 | NC_022703 | Qin et al., 2015 |
| 51 | *Takydromus sylvaticus* | 17838 | JX290083 | Tang et al., 2014 |
| 52 | *Takydromus tachydromoides* | 18245 | AB080237 | Kumazawa, 2007 |
| 53 | *Takydromus wolteri* | 18236 | NC_018777 | Yu et al., 2013 |
| 54 | *Zootoca vivipara* | 17046 | NC_026867 | Liu et al., 2015 |
| 55 | *Gekko subpalmatus* | 17105 | MK680534 | Luo et al., 2019 |
| 56 | *Gekko hokouensis* | 17769 | KT005801 | Hao et al., 2017 |

**References**

Böhme MU, Fritzsch G, Tippmann A, Schlegel M, Berendonk TU. The complete mitochondrial genome of the green lizard *Lacerta viridis viridis* (Reptilia: Lacertidae) and its phylogenetic position within squamate reptiles. Gene, 2007, 394: 69-77.

Buglione M, Petrelli S, Maselli V, Trapanese M, Salvemini M, Aceto S, Cosmo AD, Fulgione D. Fixation of genetic variation and optimization of gene expression: The speed of evolution in isolated lizard populations undergoing Reverse Island Syndrome. PLoS One, 2019, 16: e0256943.

Du Y, Qiu QB, Tong QL, Lin LH. The complete mitochondrial genome of *Eremias przewalskii* (Squamata: Lacertidae). Mitochondrial DNA Part B, 2014, 27: 1918-1919.

Hao SL, Yu DN, Ping J, Zhou HB, Zhang YP. Complete mitochondrial genomes of two gecko species, *Gekko hokouensis* and *Gekko japonicus* (Squamata, Gekkonidae). Mitochondrial DNA Part B, 2016, 1: 346-347.

Hu JG, Peng LF, Tang XS, Huang S. The complete mitochondrial genome of *Takydromus septentrionalis* (Reptilia: Lacertidae). Mitochondrial DNA Part B, 2019, 4: 2193-2194.

Kirchhof S, Lyra ML, Rodríguez A, Ineich [I](https://www.nature.com/articles/s41598-021-83422-7#auth-Ivan-Ineich), Müller J, Rödel MO, Trape JF, Vences M, Boissinot S. Mitogenome analyses elucidate the evolutionary relationships of a probable Eocene wet tropics relic in the xerophilic lizard genus *Acanthodactylus*. Scientific Reports, 2021, 11: 4858.

Komissarov A, Korchagin V, Kliver S, Dobrynin P, Semyenova S, O'Brien S, Ryskov A. The complete mitochondrial genome of the parthenogenetic Caucasian rock lizard *Darevskia unisexualis* (Squamata: lacertidae) contains long tandem repeat formed by 59 bp monomer. Mitochondrial DNA Part B, 2016, 1: 875-877.

Kumazawa Y. Mitochondrial genomes from major lizard families suggest their phylogenetic relationships and ancient radiations. Gene, 2007, 388: 19-26.

Liu P, Zhu D, Zhao WG, Ji X. The complete mitochondrial genome of the common lizard *Zootoca vivipara* (Squamata: Lacertidae). Mitochondrial DNA Part B, 2016, 27: 1944-1945.

Luo HD, Huang A, Li B, Ni QY, Yao YF, Xu HL, Zeng B, Li Y, Wei ZM, Zhang MW. Complete mitochondrial genome of the webbed-toed gecko *Gekko subpalmatus* (Squamata: Gekkonidae). Mitochondrial DNA Part B, 2019, 4: 1725-1726.

Ma WW, Liu HH, Zhao WG, Liu P. The complete mitochondrial genome of *Takydromus amurensis* (Squamata: Lacertidae). Mitochondrial DNA Part B, 2016, 1: 214-215.

Murtskhvaladze M, Tarkhnishvili D, Anderson CL, Kotorashvili A. Phylogeny of caucasian rock lizards (*Darevskia*) and other true lizards based on mitogenome analysis: optimisation of the algorithms and gene selection. PLoS One, 2020, 15: e0233680.

Podnar M, Pinsker W, Mayer W. Complete mitochondrial genomes of three lizard species and the systematic position of the Lacertidae (Squamata). Journal Zoological Systematics Evolutionary Reaearch, 2009, 47: 35-41.

Qin PS, Zeng DL, Hou LX, Yang XW, Qin XM. Complete mitochondrial genome of *Takydromus sexlineatus* (Squamata, Lacertidae). Mitochondrial DNA Part B, 2015, 26: 465-466.

Tang XS, Chen JM, Huang S. Mitochondrial genome of the Chung-an ground lizard *Takydromus sylvaticus* (Reptilia: Lacertidae). Mitochondrial DNA Part B, 2014, 25: 319-320.

Tian LL, Guo XG. Complete mitochondrial genomes of five racerunners (Lacertidae: *Eremias*) and comparison with other lacertids: insights into the structure and evolution of the control region. Gene, 2022, 13: 726.

Wang S, Liu JL, Zhang B, Guo XG. The complete mitochondrial genome of *Eremias dzungarica* (Reptilia, Squamata, Lacertidae) from the Junggar Basin in Northwest China. Mitochondrial DNA Part B, 2021, 6: 2012-2014.

Wu LX, Luo KN, Ding GH. Complete mitochondrial genome of *Takydromus kuehnei* (Squamata: *Takydromus*) and its phylogenetic analysis. Mitochondrial DNA Part B, 2022, 7: 764-765.

Yu DN, Ji X. The complete mitochondrial genome of *Takydromus wolteri* (Squamata: Lacertidae). Mitochondrial DNA Part B, 2013, 24: 3-5.

Zhou TH, Li DJ, Dujsebayeva TN, Liu JL, Guo XG. Complete mitochondrial genome of Stummer’s racerunner (*Eremias stummeri*) from Kazakhstan. Mitochondrial DNA Part B, 2016, 27: 4340-4341.
