# Supplementary material for: Climate‐driven mitochondrial selection in lacertid lizards
Source: Ecol Evol. 2024 Mar 24;14(3):e11176. doi: 10.1002/ece3.11176 (PMC10961475; doi:10.1002/ece3.11176)
Supplement: Supplementary file 3 — Table S3. [file ECE3-14-e11176-s002.doc]

Table S3 Information on mitochondrial genome size and nucleotide composition in 54 species of lacertid lizards.

|  | Species | Accession No. | mt DNA size (bp) | D-loop length (bp) | T | C | A | G | AT content | AT-skew | GC-skew |
| --- | --- | --- | --- | --- | --- | --- | --- | --- | --- | --- | --- |
| 1 | *Acanthodactylus aureus* | NC_059775 | 17021 | 1419 | 29.52 | 26.61 | 30.13 | 13.73 | 59.65 | 0.01 | -0.32 |
| 2 | *Acanthodactylus boskianus* | NC_059772 | 17143 | 1429 | 27.80 | 28.42 | 30.16 | 13.63 | 57.96 | 0.04 | -0.35 |
| 3 | *Acanthodactylus erythrurus* | NC_059773 | 16827 | 1194 | 30.23 | 25.66 | 30.67 | 13.43 | 60.90 | 0.01 | -0.31 |
| 4 | *Acanthodactylus guineensis* | NC_059781 | 16963 | 1161 | 29.56 | 25.77 | 31.07 | 13.31 | 60.63 | 0.02 | -0.32 |
| 5 | *Acanthodactylus schmidti* | NC_059782 | 16943 | 1343 | 26.81 | 28.90 | 29.98 | 14.15 | 56.79 | 0.06 | -0.34 |
| 6 | *Algyroides nigropunctatus* | NC_059780 | 15844 | 146 | 28.25 | 26.82 | 31.70 | 13.22 | 59.95 | 0.06 | -0.34 |
| 7 | *Australolacerta australis* | NC_059777 | 17019 | 1297 | 30.24 | 25.34 | 31.71 | 12.63 | 61.95 | 0.02 | -0.33 |
| 8 | *Darevskia armeniaca* | MG704915 | 17521 | 1676 | 28.54 | 26.85 | 31.92 | 12.69 | 60.46 | 0.06 | -0.36 |
| 9 | *Darevskia brauneri* | MH481137 | 16976 | 1528 | 28.34 | 26.78 | 31.80 | 13.07 | 60.14 | 0.06 | -0.34 |
| 10 | *Darevskia caucasica* | MH481131 | 16343 | 892 | 28.06 | 27.27 | 31.47 | 13.2 | 59.53 | 0.06 | -0.35 |
| 11 | *Darevskia chlorogaster* | MH481136 | 17479 | 2033 | 27.86 | 27.34 | 31.28 | 13.51 | 59.14 | 0.06 | -0.34 |
| 12 | *Darevskia clarkorum* | MH481134 | 16301 | 849 | 28.15 | 26.87 | 32.10 | 12.89 | 60.25 | 0.07 | -0.35 |
| 13 | *Darevskia daghestanica* | MH481135 | 17189 | 1733 | 28.19 | 27.03 | 31.95 | 12.83 | 60.14 | 0.06 | -0.36 |
| 14 | *Darevskia dahli* | MG704916 | 17528 | 1674 | 28.47 | 26.87 | 31.99 | 12.67 | 60.46 | 0.06 | -0.36 |
| 15 | *Darevskia derjugini* | MH481130 | 16960 | 1506 | 28.50 | 26.66 | 31.66 | 13.17 | 60.16 | 0.05 | -0.34 |
| 16 | *Darevskia mixta* | MG704917 | 17532 | 1678 | 28.45 | 26.88 | 31.98 | 12.69 | 60.43 | 0.06 | -0.38 |
| 17 | *Darevskia parvula* | MG704918 | 17510 | 1670 | 27.41 | 27.77 | 31.99 | 12.83 | 59.40 | 0.08 | -0.36 |
| 18 | *Darevskia portschinskii* | MG704919 | 17529 | 1690 | 27.56 | 27.74 | 32.02 | 12.68 | 59.58 | 0.07 | -0.37 |
| 19 | *Darevskia praticola* | MH481132 | 16418 | 975 | 27.20 | 27.81 | 31.09 | 13.90 | 58.29 | 0.06 | -0.33 |
| 20 | *Darevskia raddei* | MH481133 | 20478 | 4344 | 27.61 | 28.06 | 32.85 | 11.48 | 60.46 | 0.09 | -0.42 |
| 21 | *Darevskia rudis* | MG704920 | 17534 | 1671 | 27.61 | 27.55 | 32.22 | 12.62 | 59.83 | 0.08 | -0.37 |
| 22 | *Darevskia unisexualis* | KX644918 | 21433 | 1678 | 27.28 | 28.53 | 32.96 | 11.23 | 60.24 | 0.09 | -0.43 |
| 23 | *Darevskia valentini* | NC_045934 | 17393 | 1647 | 27.76 | 27.47 | 32.06 | 12.71 | 59.82 | 0.07 | -0.37 |
| 24 | *Eremias argus* | JQ086345 | 18521 | 3116 | 28.46 | 27.63 | 29.92 | 13.98 | 58.38 | 0.03 | -0.33 |
| 25 | *Eremias brenchleyi* | EF490071 | 19542 | 4152 | 28.30 | 27.57 | 30.12 | 14.01 | 58.42 | 0.03 | -0.33 |
| 26 | *Eremias dzungarica* | MW250881 | 19899 | 4496 | 28.41 | 26.80 | 31.63 | 13.16 | 60.04 | 0.05 | -0.34 |
| 27 | *Eremias multiocellata* | KJ664798 | 18996 | 3586 | 28.30 | 27.34 | 30.87 | 13.50 | 59.17 | 0.04 | -0.34 |
| 28 | *Eremias nikolskii* | NC_060561 | 20840 | 5436 | 28.00 | 28.01 | 30.54 | 13.45 | 58.54 | 0.04 | -0.35 |
| 29 | *Eremias przewalskii* | NC_025929 | 18825 | 2823 | 27.88 | 27.88 | 30.31 | 13.93 | 58.19 | 0.04 | -0.33 |
| 30 | *Eremias scripta* | OM935766 | 19381 | 3981 | 29.18 | 25.74 | 31.97 | 13.11 | 61.15 | 0.05 | -0.33 |
| 31 | *Eremias stummeri* | NC_029878 | 19602 | 4214 | 28.35 | 27.20 | 31.27 | 13.18 | 59.62 | 0.05 | -0.35 |
| 32 | *Eremias szczerbaki* | NC_062143 | 19650 | 4251 | 28.14 | 27.29 | 31.32 | 13.24 | 59.46 | 0.05 | -0.35 |
| 33 | *Eremias vermiculata* | KP981388 | 19796 | 4409 | 28.86 | 27.03 | 31.10 | 13.00 | 59.96 | 0.04 | -0.35 |
| 34 | *Eremias yarkandensis* | NC_060637 | 18743 | 3339 | 28.35 | 27.22 | 31.10 | 13.33 | 59.45 | 0.05 | -0.34 |
| 35 | *Gallotia atlantica* | NC_059771 | 15552 | 107 | 24.92 | 28.74 | 32.68 | 13.64 | 57.60 | 0.07 | -0.36 |
| 36 | *Lacerta agilis* | CM020436 | 19093 | 1687 | 30.09 | 25.39 | 31.61 | 12.91 | 43.00 | 0.02 | -0.33 |
| 37 | *Lacerta bilineata* | KT722705 | 17154 | 1420 | 28.28 | 27.29 | 31.25 | 13.17 | 59.80 | 0.05 | -0.35 |
| 38 | *Lacerta viridis* | NC_008328 | 17156 | 1752 | 28.45 | 26.96 | 31.32 | 13.26 | 59.77 | 0.05 | -0.34 |
| 39 | *Meroles squamulosus* | NC_059779 | 16860 | 1193 | 28.26 | 27.55 | 31.07 | 13.08 | 59.33 | 0.05 | -0.36 |
| 40 | *Mesalina olivieri* | NC_059774 | 16899 | 1175 | 28.82 | 26.69 | 31.20 | 13.27 | 60.02 | 0.04 | -0.34 |
| 41 | *Pedioplanis laticeps* | NC_059778 | 17046 | 1342 | 28.34 | 27.03 | 31.44 | 13.19 | 59.78 | 0.05 | -0.34 |
| 42 | *Phoenicolacerta kulzeri* | FJ460596 | 17199 | 1810 | 29.17 | 26.19 | 31.30 | 13.34 | 60.47 | 0.04 | -0.33 |
| 43 | *Podarcis muralis* | NC_011607 | 17311 | 1872 | 29.71 | 25.69 | 31.74 | 12.86 | 61.45 | 0.03 | -0.33 |
| 44 | *Podarcis siculus* | MH157278 | 17286 | 1832 | 29.25 | 26.34 | 31.29 | 13.13 | 60.54 | 0.03 | -0.33 |
| 45 | *Psammodromus algirus* | NC_059776 | 17118 | 1380 | 26.74 | 26.41 | 33.92 | 12.87 | 60.66 | 0.12 | -0.34 |
| 46 | *Takydromus amurensis* | NC_030209 | 17333 | 1953 | 28.80 | 26.06 | 31.23 | 13.91 | 60.03 | 0.04 | -0.30 |
| 47 | *Takydromus intermedius* | OQ632596 | 17713 | 2330 | 28.75 | 26.40 | 31.11 | 13.72 | 59.86 | 0.04 | -0.32 |
| 48 | *Takydromus kuehnei* | MZ435950 | 17224 | 1813 | 30.52 | 24.81 | 30.82 | 13.84 | 61.34 | 0.005 | -0.28 |
| 49 | *Takydromus septentrionalis* | MK630237 | 18304 | 2913 | 30.29 | 24.91 | 31.58 | 13.22 | 61.87 | 0.02 | -0.31 |
| 50 | *Takydromus sexlineatus* | NC_022703 | 18943 | 3562 | 30.79 | 24.67 | 31.48 | 13.05 | 62.27 | 0.01 | -0.31 |
| 51 | *Takydromus sylvaticus* | JX290083 | 17838 | 2452 | 29.17 | 26.02 | 31.09 | 13.72 | 60.26 | 0.03 | -0.31 |
| 52 | *Takydromus tachydromoides* | AB080237 | 18245 | 522 | 29.47 | 25.69 | 30.85 | 13.95 | 60.32 | 0.02 | -0.30 |
| 53 | *Takydromus wolteri* | NC_018777 | 18236 | 2855 | 30.69 | 24.51 | 31.31 | 13.50 | 62.00 | 0.01 | -0.29 |
| 54 | *Zootoca vivipara* | NC_026867 | 17046 | 1665 | 30.36 | 24.26 | 32.88 | 12.50 | 63.24 | 0.04 | -0.32 |
