# Supplementary material for: Climate‐driven mitochondrial selection in lacertid lizards
Source: Ecol Evol. 2024 Mar 24;14(3):e11176. doi: 10.1002/ece3.11176 (PMC10961475; doi:10.1002/ece3.11176)
Supplement: Supplementary file 4 — Table S4. [file ECE3-14-e11176-s001.doc]

table S4 Annotation of the mitochondrial genome of *T. intermedius*.

| Name | Start | Stop | Strand | Length | Intergenic nucleotides | Start codon | Stop codon | Anticodons |
| --- | --- | --- | --- | --- | --- | --- | --- | --- |
| tRNA-Phe (F) | 1 | 73 | H | 73 |  |  |  | GAA |
| 12S rRNA | 73 | 1025 | H | 953 | -1 |  |  |  |
| tRNA-Val (V) | 1025 | 1085 | H | 61 | -1 |  |  | UAC |
| 16S rRNA | 1088 | 2612 | H | 1525 | 2 |  |  |  |
| tRNA-Leu (L) | 2613 | 2685 | H | 73 | 0 |  |  | UAA |
| ND1 | 2686 | 3648 | H | 963 | 0 | ATG | TAA |  |
| tRNA-Ile (I) | 3659 | 3729 | H | 71 | 10 |  |  | GAU |
| tRNA-Gln (Q) | 3731 | 3801 | L | 71 | 1 |  |  | UUG |
| tRNA-Met (M) | 3801 | 3868 | H | 68 | -1 |  |  | CAU |
| ND2 | 3869 | 4903 | H | 1035 | 0 | ATG | TAG |  |
| tRNA-Trp (W) | 4902 | 4974 | H | 73 | -2 |  |  | UCA |
| tRNA-Ala (A) | 4977 | 5044 | L | 68 | 2 |  |  | UGC |
| tRNA-Asn (N) | 5046 | 5118 | L | 73 | 1 |  |  | GUU |
| tRNA-Cys (C) | 5148 | 5210 | L | 63 | 29 |  |  | GCA |
| tRNA-Tyr (Y) | 5216 | 5287 | L | 72 | 5 |  |  | GUA |
| COX1 | 5244 | 6833 | H | 1590 | -44 | GTG | AGG |  |
| tRNA-Ser (S) | 6829 | 6899 | L | 71 | -5 |  |  | UGA |
| tRNA-Asp (D) | 6903 | 6971 | H | 69 | 3 |  |  | GUA |
| COX2 | 6972 | 7659 | H | 688 | 0 | ATG | T |  |
| tRNA-Lys (K) | 7660 | 7723 | H | 64 | 0 |  |  | UUU |
| ATP8 | 7724 | 7785 | H | 162 | 0 | ATG | TAA |  |
| ATP6 | 7876 | 8556 | H | 681 | -92 | ATG | TAA |  |
| COX3 | 8556 | 9339 | H | 784 | -1 | ATG | T |  |
| tRNA-Gly (G) | 9340 | 9406 | H | 67 | -1 |  |  | UCC |
| ND3 | 9407 | 9754 | H | 348 | 0 | ATG | TAA |  |
| tRNA-Arg (R) | 9753 | 9819 | H | 67 | -2 |  |  | UCG |
| ND4L | 9822 | 10118 | H | 297 | 2 | ATG | TAA |  |
| ND4 | 10112 | 11492 | H | 1381 | -7 | ATG | T |  |
| tRNA-His (H) | 11493 | 11560 | H | 68 | -6 |  |  | GUG |
| tRNA-Ser (S) | 11561 | 11626 | H | 66 | 0 |  |  | GCU |
| tRNA-Leu (L) | 11626 | 11696 | H | 71 | -1 |  |  | UAG |
| ND5 | 11697 | 13523 | H | 1827 | 0 | ATG | TAA |  |
| ND6 | 13519 | 14034 | L | 516 | -5 | ATG | AGA |  |
| tRNA-Glu (E) | 14035 | 14103 | L | 69 | 0 |  |  | UUC |
| CYTB | 14106 | 15248 | H | 1143 | 2 | ATG | TAG |  |
| tRNA-Thr (T) | 15251 | 15315 | H | 65 | 2 |  |  | UGU |
| tRNA-Pro (P) | 15317 | 15384 | L | 68 | 1 |  |  | UGG |
